# Prevalence and Most Commonly Encountered QRS Fragmentation Morphology and Early Repolarization ST-Segment Changes During the Height of the COVID-19 Pandemic: A Single-Center Experience

**DOI:** 10.7759/cureus.101990

**Published:** 2026-01-21

**Authors:** Khalid Sawalha, Robert F Spraggins II, Munes Albadaineh, Andrew J Fancher, Angel Lopez Candales

**Affiliations:** 1 Cardiovascular Disease, University of Arkansas for Medical Sciences, Little Rock, USA; 2 Cardiometabolic Medicine, University of Missouri–Kansas City School of Medicine, Kansas City, USA; 3 Internal Medicine, University of Arkansas for Medical Sciences, Little Rock, USA; 4 Internal Medicine, University of Kansas School of Medicine-Wichita, Wichita, USA; 5 Cardiovascular Medicine, University of Puerto Rico School of Medicine, San Juan, USA

**Keywords:** covid-19 infection, early repolarization, fragmented qrs complex, pandemic, st-segment changes

## Abstract

Background: SARS-CoV-2 infection has been shown to cause instability of myocardial cell membranes through different mechanisms. This instability can manifest as fragmented QRS (fQRS) complexes and early repolarization (ER) on an electrocardiogram (ECG). Since ECG abnormalities during this pandemic have not been previously reported, we investigated the prevalence of fQRS and ER during the height of the COVID-19 pandemic at our institution involving two hospitals.

Methods: A total of 1,476 ECG tracings obtained between December 2020 and March 2021 at University Health-Truman Medical Centers were retrospectively reviewed by a single cardiologist. Current and prior tracings were analyzed for ER changes, and fQRS was identified using the traditional Das criteria. Patients were further stratified by age, race, and gender.

Results: Of the 1,476 patients, 570 (39%) had fQRS and 68 (4.6%) had ER changes. Respective demographics, associated rhythms, and presence of left ventricular hypertrophy were also reported. Among patients with fQRS, we report the most common ECG lead locations and morphologies.

Conclusion: In our single-institution study, ECGs obtained during the peak of the COVID-19 pandemic showed a prevalence of 39% for fQRS and 4.6% for ER. While the prevalence of ER was comparable to that observed in the general population, newly identified fQRS was observed at a substantially higher frequency than has been reported in healthy adult populations.

## Introduction

Identification of fragmentation of QRS complexes (fQRS), as seen on routine surface electrocardiography (ECG) examinations, was initially described in individuals with left ventricular hypertrophy and patients with a myocardial scar after myocardial infarction [1]. However, emerging data have broadened the findings of these fQRS ECG abnormalities in several other cardiac as well as non-cardiac clinical entities [2]. More importantly, the presence of fQRS has been linked to adverse cardiac events and increased mortality [3]. 

In healthy adults, fQRS on routine 12-lead ECG appears to be a normal variant with a prevalence of 5.1% and a predominant distribution in inferior leads [4]. However, the prevalence of fQRS during the height of the pandemic has never been reported.

Similarly, different definitions have been used over the years to define early repolarization (ER) ECG abnormalities; the most recent consensus identifies variants that can be useful in risk stratification regarding potential for either malignant arrhythmic events or risk of sudden cardiac death (SCD) [5]. Unfortunately, estimates of ER have been described as “disconcerting” because of reported prevalence ranging from 2% to 31% [5]. 

The novel coronavirus, previously designated 2019-nCoV, was identified in Wuhan, China at the end of 2019. It subsequently spread worldwide, becoming a global health pandemic. This virus, severe acute respiratory syndrome coronavirus 2 (SARS-CoV-2), is the cause of COVID-19 infection. Systemic SARS-CoV-2 infection has been associated with several cardiovascular complications, including myocardial injury and myocarditis, acute myocardial infarction, heart failure, dysrhythmias, myocardial fibrosis, and venous thromboembolic embolism due to the excessive production of pro-inflammatory cytokines [6].

Since myocardial scar and myocarditis have been linked to COVID-19, and both complications are associated with fQRS and ER, we analyzed ECG tracings from the height of the COVID-19 pandemic at University Health-Truman Medical Centers with the hypothesis of increased prevalence of these ECG abnormalities.

## Materials and methods

Study design and population

This study was designed as a retrospective observational analysis of standard 12-lead electrocardiograms (ECGs). A total of 1,476 ECG tracings were randomly selected from adult patients evaluated at University Health-Truman Medical Centers, Kansas City, Missouri, between December 2020 and March 2021. Tracings that flowed into the MUSE (GE Healthcare) ECG management system were chosen using a non-systematic method from all eligible adult 12-lead ECGs. Both index and available prior ECGs for each patient were reviewed to assess for the presence of fQRS and ER patterns. Demographic variables collected for analysis included age, self-reported race, and sex.

Ethical approval

The study protocol was reviewed and approved by the University Health Institutional Review Board. Given the retrospective nature of the study and the use of de-identified clinical data, the requirement for written informed consent was waived.

ECG acquisition and interpretation

All ECGs were retrieved from the MUSE ECG management system (GE Healthcare, Chicago, Illinois). Recordings were obtained using standard clinical 12-lead ECG acquisition protocols. ECGs were recorded at a paper speed of 25 mm/s (occasionally 50 mm/s) and a standard calibration of 10 mm/mV. The default institutional filter settings were applied, including a high-pass filter of 0.05-0.15 Hz, a low-pass filter of 100-150 Hz, and an alternating current filter of 50 or 60 Hz. No additional post-processing or signal modification was performed for study purposes [4].

All ECGs were interpreted by a single board-certified cardiologist to minimize inter-observer variability. The reviewer was blinded to patient clinical outcomes at the time of ECG assessment.

Definition of fragmented QRS

fQRS complexes were identified according to the criteria originally described by Das et al [4]. fQRS was defined by the presence of an additional R wave (R′), notching in the nadir of the R wave or S wave, or the presence of more than one R′ (fragmentation) within the QRS complex, occurring in at least two contiguous leads. To avoid confounding due to abnormal ventricular conduction, ECGs demonstrating complete or incomplete left or right bundle branch block patterns, defined as a QRS duration ≥120 ms, were excluded from the fQRS analysis [4].

Definition of early repolarization

ER was defined in accordance with the consensus criteria published by Macfarlane et al. QRS duration was required to be <120 ms. ER was identified by the presence of a J-point elevation (Jp) ≥0.1 mV in at least two contiguous leads, excluding leads V1 through V3. Morphologic criteria included an end-QRS notch or slur located on the downslope of a prominent R wave. For notched patterns, the notch was required to lie entirely above the isoelectric baseline; for slurred patterns, the onset of the slur was required to be above the baseline [5].

Data analysis

ECGs were categorized based on the presence or absence of fQRS and ER patterns. Patients were subsequently stratified by age, race, and sex for descriptive and comparative analyses. No therapeutic interventions were performed as part of this study.

## Results

Among the 1476 ECGs reviewed, the fQRS ECG abnormality was seen in 570 patients (39%) and ER changes were present in 68 patients (4.6%). As shown in Table 1, the mean age of patients with fQRS was 50 ± 15 years, while those with ER had a mean age of 49 ± 16 years. Gender distribution was equal for fQRS patients (50% male and 50% female), whereas ER was predominantly seen in male patients (78%). Race data revealed that fQRS was most prevalent among black individuals (57%), similar to ER with (78%) of black patients. Normal sinus rhythm was the most frequent in both groups with 72% in the fQRS group and 78% in the ER group. Left ventricular hypertrophy (LVH) was notably higher in the ER group at 27% compared with only 11% in the fQRS group. Localization of the fQRS abnormalities is detailed in Table 2, with the inferior leads being the most affected, with (20%) in lead III, (13%) in lead III and aVF, and 7% in lead aVF. Morphological patterns of fQRS are summarized in Table 3. The most frequent morphology was a notched R peak (38.9%), followed by a notched R upper descending R wave (28.1%), and a notched R ascending R wave (19.6%). Less common patterns included notched R in the lower descending R wave, incomplete right bundle branch block, and various rSR variants.

**Table 1:** Baseline characteristics of the study population. *Race distribution in the fQRS dataset was available for 305 patients (subset) and for 40 patients in the ER dataset. fQRS: Fragmented QRS; ER: early repolarization; LVH: left ventricular hypertrophy

Variable		fQRS (n = 570/1476)	ER (n = 68/1476)
Age, mean ± SD (years)		50 ± 15	49 ± 16
Gender	Male sex, n (%)	287 (50%)	52 (78%)
	Female sex, n (%)	283 (50%)	15 (22%)
Race	Black, n (%)	175 (57% of 305*)	31 (78% of 40*)
	White, n (%)	96 (31% of 305*)	5 (13% of 40*)
	Hispanic, n (%)	34 (11% of 305*)	4 (10% of 40*)
Rhythm, n (%)	Normal sinus rhythm, n (%)	408 (72%)	52 (78%)
	Sinus tachycardia, n (%)	108 (19%)	8 (12%)
	Sinus bradycardia, n (%)	48 (8%)	7 (10%)
	Supraventricular tachycardia, n (%)	1 (0.2%)	—
	Atrial fibrillation, n (%)	5 (0.9%)	—
LVH present, n (%)		63 (11%)	18 (27%)

**Table 2:** Location of the fQRS abnormalities fQRS: Fragmented QRS

Localization of fQRS	Percent of Patients Affected (n=Number of Patients)
aVF	7% (n=40)
aVF and precordial lead combo	2% (n=11)
aVL	16% (n=91)
aVL and precordial lead combo	2% (n=11)
aVR	0.4% (n=2)
aVR and precordial lead combo	0.2% (n=1)
Lead I	0.5% (n=3)
Limb leads combo	7% (n=40)
Multiple limb and precordial lead combo	3% (n=17)
Lead III	20% (n=114)
III and aVF	13% (n=74)
III, aVF and single V1	2% (n=11)
III, aVF and two precordial leads	3% (n=17)
III and aVL	9% (n=51)

**Table 3:** Morphological patterns of fQRS fQRS: Fragmented QRS; RBBB: right bundle branch block

fQRS pattern	Percent of Patients Affected (n=Number of Patients)
Notched R peak	38.9% (n=222)
Notched R upper descending R wave	28.1% (n=160)
Notched R ascending R wave	19.6% (n=112)
Notched R lower descending R wave	5.1% (n=29)
Incomplete RBBB	1.6% (n=9)
rSR	1.4% (n=8)
rSr’	1.4% (n=8)
RSr’	1.1% (n=6)
RSR’	0.9% (n=5)
Multiple spikes	0.2% (n=1)

## Discussion

To the best of our knowledge, we present the first prevalence data on both fQRS and ER based on a retrospective analysis of routine ECGs randomly obtained during the height of the COVID-19 pandemic.

With regard to fQRS, traditionally considered a potential marker of myocardial scar, we report a prevalence of 39%. This prevalence exceeds the pre-pandemic rate of 5.1% and is comparable to that observed in patients with prior myocardial infarction (39.5%) [7,8]. The abnormality was more prevalent in Black patients and most frequently observed in the inferior limb leads. The most common fQRS patterns encountered were notched ascending R waves, notched R peaks, and notching of the upper and lower descending limbs of the R wave. These findings are consistent with the literature, which identifies the most frequent fQRS pattern as either an additional R′ (rsR′ or rSr′ type) or notching in the nadir of the S wave [6].

ER has historically been considered a benign finding without clinical significance. The recent emergence of several case reports, case-control studies, and population studies has linked ER as a potential risk factor for fatal arrhythmia, particularly ventricular fibrillation, and SCD [9-12]. Our data shows a prevalence of 4.6% during the COVID-19 pandemic, which is not considerably different from the prevalence reported pre-pandemic. Depending on the source, estimates of ER have ranged from 2% to 31% [8] and are mostly seen among younger patients [11-13]. In our population, 51% of patients had previous ECGs with similar ER changes. Therefore, our data suggests that the prevalence of ER ECG changes has not significantly changed with the pandemic.

Acute respiratory distress syndrome and acute hypoxic respiratory failure have been the main complications among COVID-19 patients. However, excessive production of pro-inflammatory cytokines produced during infection has been associated with several cardiovascular complications including myocardial injury, myocarditis, acute myocardial infarction, heart failure, dysrhythmias, myocardial fibrosis, and venous thromboembolic embolism [14-16]. The underlying pathophysiologic mechanisms and their consequences become more relevant given our findings of increased fQRS prevalence with the pandemic.

After our data was acquired and analyzed, other studies have utilized surface ECGs to identify markers of adverse prognosis in patients hospitalized with COVID-19 infection. Specifically, fQRS and ER were assessed in COVID-19 patients to identify patients at risk for adverse prognosis [17-27]. While these studies were useful to identify relationships between fQRS and ER ECG changes and adverse cardiac outcomes, we present data on a much larger patient sample during the peak of the COVID-19 pandemic and offer insight into how the prevalence of these ECG abnormalities has changed. Additionally, our data includes insight into lead location prevalence and most commonly encountered fQRS patterns, associated cardiac arrhythmias, and presence of LVH which have not been described in this population with these ECG abnormalities.

We do acknowledge the following limitations. First, this is a single-center experience with two hospitals. These findings would be better supported through a large multi-center prospective and collaborative study. Second, although this analysis occurred during the peak of the COVID-19 pandemic, we cannot reasonably say all patients were previously or currently infected with COVID-19, as not all patients were tested for infection at the time the ECG was obtained. Thus, there is no control group to compare results. The main objective was to assess this prevalence during the COVID-19 pandemic, as it was not previously reported.

## Conclusions

In summary, the prevalence observed in this randomly selected cohort at our institution exceeds pre-pandemic reported rates. These findings generate hypothesis-forming signals and justify further investigation incorporating confirmed COVID-19 status, longitudinal follow-up, and adjudicated hard cardiac endpoints, including arrhythmic events. It would also be clinically relevant to determine whether these ECG abnormalities regress following recovery from COVID-19 infection. Although arrhythmic risk was a major concern during the pandemic, our data suggest that the prevalence of ER patterns remained comparable to pre-pandemic reports.

These results must be interpreted in light of important limitations such as that this is a retrospective, single-center experience across two hospitals, limiting generalizability and highlighting the need for larger, prospective, multicenter studies. Accordingly, the primary intent of this study was to describe ECG pattern prevalence during the pandemic period, an area for which prior data were lacking.
